# TAK1 regulates resident macrophages by protecting lysosomal integrity

**DOI:** 10.1038/cddis.2017.23

**Published:** 2017-02-09

**Authors:** Yosuke Sakamachi, Sho Morioka, September R Mihaly, Giichi Takaesu, Julie F Foley, Michael B Fessler, Jun Ninomiya-Tsuji

**Affiliations:** 1 Department of Biological Sciences, North Carolina State University, Raleigh, NC 27695-7633, USA; 2 Cellular and Molecular Pathology Branch, National Institute of Environmental Health Sciences, National Institutes of Health, Research Triangle Park, NC 27709, USA; 3 Immunity, Inflammation and Disease Laboratory, National Institute of Environmental Health Sciences, National Institute of Health, Research Triangle Park, NC 27709, USA

## Abstract

Hematopoietic cell survival and death is critical for development of a functional immune system. Here, we report that a protein kinase, TAK1, is selectively required for resident macrophage integrity during embryogenesis. Hematopoietic lineage-specific deletion of *Tak1* gene (Tak1^HKO^) caused accumulation of cellular debris in the thymus in perinatal mice. Although no overt alteration in thymocytes and blood myeloid populations was observed in Tak1^HKO^ mice, we found that thymic and lung macrophages were diminished. In the *in vitro* setting, *Tak1* deficiency caused profound disruption of lysosomes and killed bone marrow-derived macrophages (BMDMs) without any exogenous stressors. Inhibition of the lysosomal protease, cathepsin B, partially blocked *Tak1*-deficient BMDM death, suggesting that leakage of the lysosomal contents is in part the cause of cell death. To identify the trigger of this cell death, we examined involvement of TNF and Toll-like receptor pathways. Among them, we found that deletion of *Tnfr1* partially rescued cell death. Finally, we show that *Tnfr1* deletion partially restored thymic and lung macrophages *in vivo*. These results suggest that autocrine and potentially paracrine TNF kills *Tak1*-deficient macrophages during development. Our results reveal that TAK1 signaling maintains proper macrophage populations through protecting lysosomal integrity.

Macrophages possess unique functional roles that are dependent on the microenvironment where they reside.^1^ During early embryogenesis, progenitors of macrophages migrate to several different sites in the embryo, uniquely differentiate, and colonize as tissue-resident macrophages, such as microglia, thymic macrophages and pulmonary macrophages.^2^ When invasion of pathogenic microorganisms occurs, tissue-resident macrophages along with recruited circulating monocytes provoke inflammatory responses. In addition to their role as responders to insult, tissue-resident macrophages are also critical for tissue integrity in the steady state. Because tissues constantly produce or acquire dead cells, cell debris, and excess lipoproteins, which belong to the family of damage-associated molecular patterns (DAMPs), tissues can become spontaneously inflamed through activation of DAMP receptors such as Toll-like receptors (TLRs).^3^ Resident macrophages are responsible for clearing DAMPs and preventing unnecessary inflammation. Thus, investigations to delineate the mechanisms by which tissue-resident macrophages differentiate and are maintained are critical for a fundamental understanding of tissue homeostasis. The mechanisms by which tissue-resident macrophages are uniquely differentiated toward specific types have begun to be elucidated.^1, 4^ However, although it is clear that tissue-resident macrophages are maintained separately from the bone marrow hematopoietic system, how their population is regulated or maintained is still largely undetermined.

TAK1 (MAP3K7) is a member of the mitogen-activated protein kinase kinase kinase (MAP3K) family, and is activated by a diverse set of inflammatory stimuli including inflammatory cytokines, TNF and IL-1, and TLR ligands.^5^ In mouse models, tissue-specific deletion of the *Tak1* gene causes cell death resulting in tissue injury in multiple tissues including the epidermis, the intestinal epithelium, and the vascular endothelium.^6, 7, 8^ A profound increase of reactive oxygen species (ROS) is causally associated with *Tak1* deficiency-induced cell death.^9, 10, 11^ The mechanism by which *Tak1* deficiency disrupts cellular redox homeostasis is not yet fully understood; however, impairment of several intracellular signaling cascades and transcription factors including but not limited to NF-*κ*B, MAPKs including p38 and JNK, and the antioxidant transcription factor Nrf2 has been implicated in increased ROS.^5, 12^ Interestingly, recent studies have revealed that not all tissues or cell types are damaged or killed by *Tak1* deletion; for example, *Tak1*-deficient neurons do not exhibit any abnormalities,^13^ and some hematopoietic cells seem to be highly resistant to *Tak1* deficiency as discussed below. Thus, sensitivities to *Tak1* deficiency vary depending on cell type and the cellular context. It still remains to be determined which cell types are sensitive to *Tak1* deficiency and the mechanism(s) by which *Tak1*-deficient cells die.

In the hematopoietic system, TAK1 is involved in maintenance of several specific cell types. We previously demonstrated that *Tak1* deficiency impairs adult hematopoietic stem cell (HSC) maintenance.^14^ Competitive transplantation assays showed that *Tak1*-deficient bone marrow cells in adult mice are incapable of repopulating any types of hematopoietic cells.^14, 15^ In contrast, T or B cell-specific deletion of *Tak1* skews subset populations of T and B cells but does not cause overt cell death in the *in vivo* setting.^16, 17, 18, 19^ Mice with myeloid-specific deletion of *Tak1* were generated and characterized by two groups using the *LysM-Cre* deleter strain.^20, 21^ These mice exhibit an increased circulating neutrophil population and develop splenomegaly, and no overt increase in cell death is observed *in vivo*.^20, 21^ However, as the *LysM-Cre* system is not highly effective in several types of myeloid cells including resident macrophages^22, 23, 24^ and its inefficient recombination of floxed genes is known to cause artificial consequences,^25^ the role of TAK1 in hematopoietic cells should be further evaluated in other gene deletion systems.

In the current study, we investigated how TAK1 participates in the hematopoietic system by using the *Vav-Cre* system, which is expressed in all the hematopoietic compartments.^26^ Specifically, *Vav1* (a GDP/GTP nucleotide-exchange factor for Rho/Rac) is expressed in erythro-myeloid progenitors and fetal HSCs that originate in the yolk sac during early embryogenesis, presumably starting around embryonic day 7.^27^ Endothelial cells also originate from the hemogenic endothelial cells.^28, 29^ However, *Vav1* is expressed only in erythro-myeloid progenitors and fetal HSCs but not in endothelial cells, and *Vav-Cre* is thus suitable for the characterization of target genes in the embryonic hematopoietic system without affecting the endothelium. Furthermore, as erythro-myeloid progenitors give rise to tissue-resident macrophages, this system provides the means to characterize target genes in tissue-resident macrophages.^22^

## Results

### Hematopoietic-specific *Tak1* deficiency impairs clearance of dead cells and causes perinatal lethality

TAK1 is required for proper development of the hematopoietic system and maintenance of adult HSC.^14, 15, 16, 17, 18, 19, 20, 21^ However, our understanding of the roles of TAK1 in various hematopoietic compartments, including those during embryogenesis, remains incomplete. *Tie2* (an angiopoietin receptor)*-Cre* system, can recombine floxed genes during early embryogenesis in hemogenic endothelial cells, early precursors of hematopoietic cells,^30^ and is suitable for investigations of the entire hematopoietic system. However, as endothelial cells share the same origin, *Tie2-Cre* recombines floxed genes in endothelial cells. We previously demonstrated that *Tak1* deletion with the *Tie2-Cre* deleter causes endothelial cell death and early embryonic lethality at embryonic day (E)10.5.^6^ We showed that erythrocytes are normally developed but blood vessel regression occurs at E10.5 in these mice, suggesting that endothelial cell autonomous defects but not hematopoietic abnormality cause blood vessel regression.^6^

**Figure 1:**
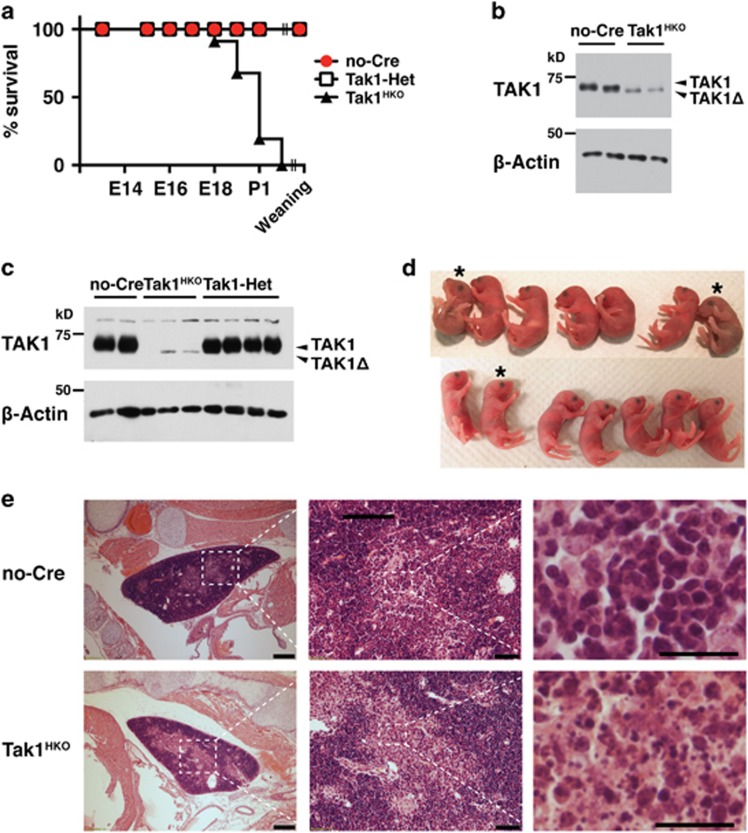
Hematopoietic-specific *Tak1* deficiency causes perinatal lethality. (**a**) Percentages of viable no-Cre (*Tak1*^flox/flox^ or *Tak1*^flox/+^), Tak1-Het (*vav-Cre Tak1*^flox/+^), and Tak1^HKO^ (*vav-Cre Tak1*^flox/flox^) embryos. The total numbers of animals analyzed including live and dead were no-Cre, 178; Tak1-Het, 110, Tak1^HKO^, 94. *P*<0.001 (log-rank test). (**b**) no-Cre control and Tak1^HKO^ thymus were isolated from E16.5 mice and analyzed by immunoblotting for the indicated targets. Recombined *Tak1* gene expressed a truncated and non-functional TAK1 protein (TAK1Δ). *β*-actin is shown as a loading control. (**c**) no-Cre, Tak1-Het, and Tak1^HKO^ blood were isolated from E18.5 mice and analyzed by immunoblotting for the indicated targets. (**d**) P0 littermates from the mating of *Tak1*^flox/flox^and *Tak1*^flox/+^ *Vav-Cre* parents. Asterisks indicate Tak1^HKO^ mice. (**e**) H&E staining of E18.5 no-Cre and Tak1^HKO^ thymus. Scale bars, 200 *μ*m (left panels), 50 *μ*m (middle panels), 20 *μ*m (right panels)

In the current study, we sought to determine the role of TAK1 specifically in the hematopoietic compartment. *Vav-Cre* was chosen as it is expressed in erythro-myeloid progenitors and fetal HSCs but not in endothelial progenitors.^27, 31^ We generated *Vav-Cre Tak1*^flox/flox^ (Tak1^HKO^) mice and compared them with littermate Cre-negative *Tak1*^flox/flox^ and heterozygous *Tak1* deletion *Vav-Cre Tak1*^flox/+^ mice. Tak1^HKO^ mice were found to suffer lethality between E18.5 and postnatal (P) day 1 (Figure 1a). We confirmed that intact TAK1 protein was diminished in all hematopoietic cells including thymocytes at E16.5 (Figure 1b) and in circulating blood cells at least by E18.5 (Figure 1c). We note here that *Tak1*-floxed gene expresses a truncated and non-functional form of TAK1 protein (TAK1Δ) when *loxP* recombination occurs, and TAK1Δ is less stable compare to intact TAK1.^8, 17^ Heterozygous deletion of *Tak1* did not observably reduce the TAK1 protein level, suggesting that a single allele is sufficient for maintenance of the steady-state TAK1 protein level. Consistently, we did not observe any abnormality in *Vav-Cre Tak1*^flox/+^ (Tak1-Het) mice, as we also observed no abnormality in other tissue-specific heterozygous *Tak1*-deficient mice.^6, 7, 8, 32^

Although Tak1^HKO^ mice die around birth, we did not observe any overt clinical abnormalities except occasional mild cyanosis (Figure 1d). We performed histological analyses of tissue samples from live Tak1^HKO^ and their littermate mice at P0. The prominent observable abnormality was found in the thymic medulla, where there was an accumulation of marked dead cell debris (Figure 1e, Supplementary Figures S1 and S2). We also note that the size of lung alveoli in Tak1^HKO^ animals was reduced compared with that in the littermate controls (Supplementary Figure S3), which may be associated with cyanosis. By contrast, other organs were architecturally indistinguishable from those in control littermates (Supplementary Figure S3). We also performed histopathological evaluation of one E18.5 litter consisting of three Tak1^HKO^ and three control embryos. At E18.5, no abnormality in the overall morphogenesis of Tak1^HKO^ embryos was observed, indicating that hematopoietic TAK1 is not required for embryogenesis. No increase in apoptotic or necrotic cells was observed in the liver, intestine, kidney, heart, spleen, lung, brain and spleen. Thus, hematopoietic-specific *Tak1* deletion does not impair normal morphogenesis or increase of cell death during embryogenesis, but causes a destruction of thymus and an abnormality in the lung architecture around birth.

### Hematopoietic-specific deletion of *Tak1* does not impair normal development of lymphocytes and myeloid cells

**Figure 2:**
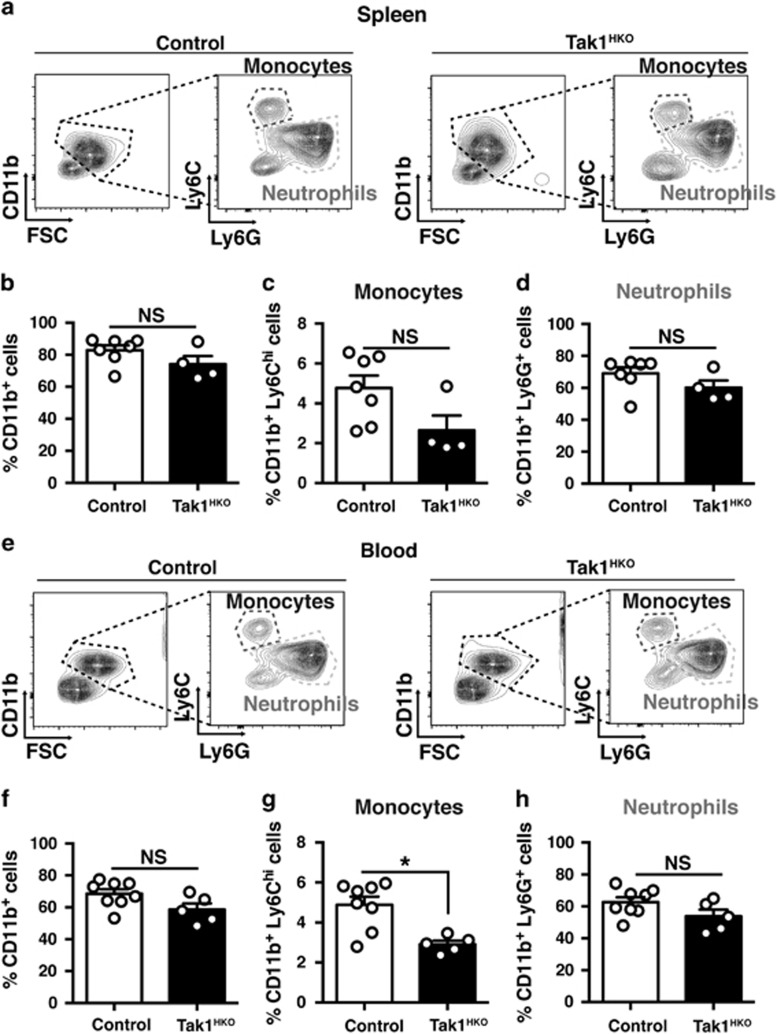
Hematopoietic-specific *Tak1* deletion does not impair the development of splenocytes and circulating myeloid cells. (**a**) Gating strategy and representative flow cytometry data of control (no-Cre and Tak1-Het) and Tak1^HKO^ splenocytes at P0. (**b**) Total CD11b^+^ cells in P0 control (*n*=7) and Tak1^HKO^ (*n*=4) spleen as a percent of total live cells. (**c**) CD11b^+^ Ly6C^hi^ cells (monocytes) and (**d**) CD11b^+^ Ly6G^+^ cells (neutrophils) in P0 control (*n*=7) and Tak1^HKO^ (*n*=4) spleen as a percent of total live cells. (**e**) Gating strategy and representative flow cytometry data of control (no-Cre and Tak1-Het) and Tak1^HKO^ blood at P0. (**f**) Total CD11b^+^ cells in P0 control (*n*=8) and Tak1^HKO^ (*n*=5) blood as a percent of total live cells. (**g**) CD11b^+^ Ly6C^hi^ cells (monocytes) and (H) CD11b^+^ Ly6G^+^ cells (neutrophils) in P0 control (*n*=8) and Tak1^HKO^ (*n*=4) blood as a percent of total live cells. Means±S.E. and all data points are shown. *P<0.05; NS, not significant (two-tailed Student's *t*-test with equal distributions)

To determine the cause of Tak1^HKO^ thymus abnormality, we first asked if *Tak1* deficiency causes any abnormalities in hematopoietic cell compartments within the systemic immune system. We began by analyzing leukocytes in circulating blood and spleen isolated from live P0 Tak1^HKO^ and littermate control mice (Figure 2). No difference in the proportions of CD11b^+^ (myeloid) and CD11b^−^ (non-myeloid, including lymphoid) cells was observed between *Tak1*-deficient mice and controls in the circulating blood or the spleen (Figures 2a, b, e and f). Among myeloids, CD11b^+^ Ly6C^hi^ (monocytes) and CD11b^+^ Ly6G^+^ (neutrophils) were similarly observed in the spleen and blood of Tak1^HKO^ and controls mice (Figures 2a and e). Although Tak1^HKO^ exhibited a trend of reduced myeloid population (Figures 2c and g), neutrophils were not altered by *Tak1* deletion. These results demonstrate that TAK1 is dispensable for normal hematopoietic development of both myeloid and lymphoid cells in the spleen and circulating blood at least around birth in mice with exception of a slight decrease in monocyte population. Hence, the thymic abnormality in Tak1^HKO^ is not caused by defects in the systemic hematopoiesis.

### Hematopoietic-specific deletion of *Tak1* diminishes thymic and pulmonary macrophages

**Figure 3:**
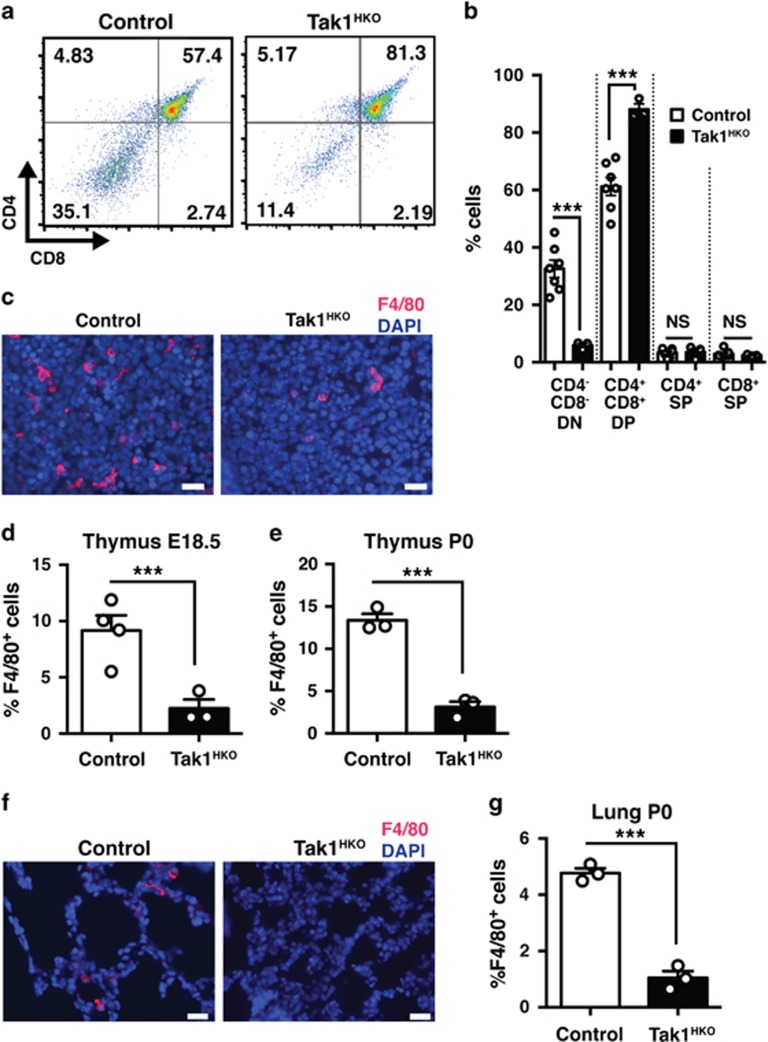
Hematopoietic-specific deletion of *Tak1* diminishes thymic and pulmonary macrophages. (**a**) Thymocytes were isolated from E18.5 control (no-Cre and Tak1-Het, *n*=7) and Tak1^HKO^ (*n*=3), and analyzed by flow cytometry. Gating strategy and representative data of control and Tak1^HKO^ including percent of cells in each quadrant are shown. (**b**) Quantification of percent DN, CD4^−^ CD8^−^; DP, CD4^+^ CD8^+^; SPCD4, CD4^+^ CD8^−^; and SPCD8, CD4^−^ CD8^+^ thymocytes of samples shown in **a**. (**c**) E18.5 control and Tak1^HKO^ thymus was analyzed by immunofluorescence staining using anti-F4/80 antibody (red) and DAPI (blue). Scale bars, 20 *μ*m. (**d**) Quantification of F4/80^+^ cells in in total DAPI stained cells of the thymus from E18.5 control (*n*=4) and Tak1^HKO^ (*n*=3). (**e**) P0 thymus control (*n*=3) and Tak1^HKO^ (*n*=3). (**f**) P0 control and Tak1^HKO^ lung was analyzed by immunofluorescence staining using anti-F4/80 antibody (red) and DAPI (blue). Scale bars, 20 *μ*m. (**g**) Quantification of F4/80^+^ cells in total DAPI stained cells. P0 control (*n*=3); Tak1^HKO^ (*n*=3). Means±S.E. and all data points are shown. ***P<0.001; NS, not significant (two-tailed Student's *t*-test with equal distributions)

We next focused on the thymus. Accumulation of cell debris might be caused by increased thymocyte death during T-cell maturation in the thymus. We thus analyzed T-cell populations in the thymus. If T cells were dying during maturation, the number of CD4^+^ CD8^+^ double positive and CD4^+^ CD8^−^ and/or CD4^−^ CD8^+^ single positive cells should be decreased by *Tak1* deficiency. Unexpectedly, we found increased CD4^+^ CD8^+^ double positive cells in Tak1^HKO^ thymus, whereas the CD4^−^ CD8^−^ double negative population was decreased (Figures 3a and b). The numbers of mature CD4^+^ CD8^−^ and CD4^−^ CD8^+^ single positive cells were comparable between control and *Tak1*-deficient thymus (Figures 3a and b). Thus, *Tak1* deficiency does not impair T-cell development and maturation. The CD4^−^ CD8^−^ double negative populations contain both naive T cells and other cell types in the thymus including thymic macrophages. Thymic macrophages have an indispensable role in clearance of dead thymocytes during T-cell development.^33^ Thus, *Tak1* deficiency might impair thymic macrophages, which could cause accumulation of cell debris in the thymus. To test this possibility, we analyzed F4/80^+^ macrophages in the Tak1^HKO^ and control thymus. The number of thymic macrophages was markedly reduced by *Tak1* deficiency (Figures 3c–e), indicating that TAK1 is required for establishment and/or maintenance of thymic macrophages. This suggested the possibility that TAK1 also contributes to integrity of other tissue-resident macrophages. We analyzed F4/80^+^ macrophages in the lung and spleen of P0 Tak1^HKO^ and control mice. The number of macrophages was also markedly reduced in the Tak1^HKO^ lung and spleen compared with the controls (Figures 3f, g and Supplementary Figure S4). These results demonstrate that TAK1 is required for establishment and/or maintenance of resident macrophages in the thymus, lung and spleen.

### *Tak1* deficiency impairs lysosomes and kills bone marrow-derived macrophages without exogenous stressors

**Figure 4:**
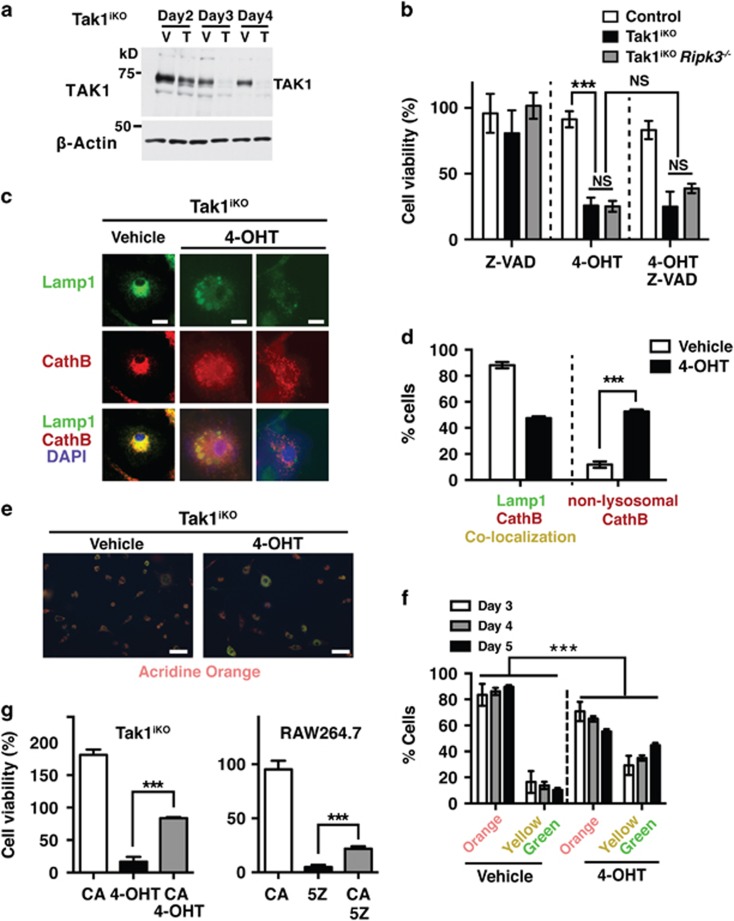
*Tak1* deficiency impairs lysosomes and kills BMDMs. (**a**) Inducible *Tak1*-deficient (Tak1^iKO^) BMDM treated with vehicle alone (ethanol, V) or 0.3 *μ*M 4-OHT (T) for 2, 3 and 4 days and analyzed by immunoblotting for TAK1. *β*-actin is shown as a loading control. (**b**) Measurement of percent cell viability of control (*n*=3), Tak1^iKO^ (*n*=3), and Tak1^iKO^ *Ripk3*^−/−^ (*n*=4) BMDMs 5 days post 0.3 *μ*M 4-OHT treatment using crystal violet viability assay. Some cells were incubated with Z-VAD-FMK (Z-VAD, 20 *μ*M) for 3 days prior to fixation. (**c** and **d**) Tak1^iKO^ BMDMs were treated with vehicle or 0.3 *μ*M 4-OHT for 5 days. Lysosomal architecture disruption was observed in Tak1^iKO^ BMDMs 5 days post 0.3 *μ*M 4-OHT treatment. Lysosomal architecture was visualized by staining using anti-lamp1 (green) and anti-cathepsin B (CathB; red) antibodies. Scale bars, 10 *μ*m. (**e** and **f**) Tak1^iKO^ BMDMs were treated with vehicle or 0.3 *μ*M 4-OHT. Lysosomal function was determined by incubating cells in acridine orange at 3, 4 and 5 days post 4-OHT treatment. Orange staining indicates normal functional lysosomal pH (around 3.5), and yellow or green staining indicates increased lysosomal pH. Scale bars, 50 *μ*m. (**g**, Left) Viability of Tak1^iKO^ BMDMs treated 30 *μ*M CA-074Me (cathepsin B inhibitor, CA), 0.3 *μ*M 4-OHT, or 30 *μ*M CA+0.3 *μ*M 4-OHT. Percent live Tak1^iKO^ cells relative to the vehicle-treated same genotype cells are shown. (Right) RAW264.7 cells were treated with 30 *μ*M CA, 200 nM 5Z-7-oxozeaenol (5Z), or 30 *μ*M CA+200 nM 5Z. All graphs, means±S.D.; ***P<0.001; NS, not significant (one-way ANOVA)

We next attempted to determine the mechanism by which *Tak1* deficiency reduces resident macrophages. It has previously been reported that *Tak1*-deficient bone marrow-derived macrophages (BMDM) spontaneously undergo cell death.^20, 34, 35^ Thus, loss of viability by *Tak1* deficiency is likely to be the cause of reduced resident macrophages. However, the mechanism by which *Tak1* deficiency spontaneously kills macrophages is still elusive. To gain mechanistic insights, we took advantage of the inducible gene deletion system. We prepared BMDM from mice having the ubiquitous inducible Cre deleter (*Rosa26-CreERT*)^36^ and *Tak1*-floxed genes (Tak1^iKO^). TAK1 was diminished in Tak1^iKO^ BMDMs after 3 days treatment of 4-hydroxytamoxifen (4-OHT) (Figure 4a). *Tak1*-deficient cells including fibroblasts and keratinocytes die with apoptosis, and RIPK3-dependent necroptosis has also been implicated in cell death by *Tak1* deficiency when *Tak1*-deficient cells are treated with a pan-caspase inhibitor, Z-VAD-FMK (Z-VAD).^5^ However, we unexpectedly found that *Tak1*-deficient BMDM death could not be rescued by either single or combined inhibition of caspases (apoptosis) and/or RIPK3, a mediator of necroptosis (Figure 4b). Thus, *Tak1*-deficient macrophages die primarily not through either apoptosis or necroptosis.

We explored other modes of cell death, including pyroptosis and lysosome malfunction-induced cell death. The pan-caspase inhibitor Z-VAD-FMK, which inhibits the pyroptosis mediator caspase 1 and caspase 11,^37^ did not block *Tak1*-deficient BMDM death (Figure 4b), suggesting that pyroptosis is not the major cause of cell death. Notably, however, Tak1^iKO^ BMDMs exhibited abnormal lysosomal architecture (Figure 4c), and co-localization of the lysosomal protease, cathepsin B and a lysosome marker, lamp1, was disrupted (Figures 4c and d). Furthermore, acridine orange staining revealed that *Tak1* deficiency increased dysfunctional lysosomes with an elevated pH (yellow and green) (Figures 4e and f). Such abnormalities in lysosomes were not observed in 4-OHT treated BMDMs having a different floxed gene together with *Rosa26-CreERT* (Supplementary Figure S5), indicating that neither 4-OHT nor Cre are the cause of the lysosome abnormalities. Thus, *Tak1* deficiency is the cause of lysosomal abnormality. We asked whether inhibition of a lysosomal protease, cathepsin B, could restore cell death in Tak1^iKO^ BMDMs. Inhibition of cathepsin B increased cell number even in wild type BMDMs about 2-fold (Figure 4g, left graph), suggesting that inhibition of cathepsins generally improves BMDM survival and/or proliferation. Importantly, inhibition of cathepsin B increased the number of Tak1^iKO^ BMDMs with higher efficiency (3-fold) than wild type BMDMs, suggesting that cathepsin activity is responsible, at least in part, for Tak1^iKO^ BMDM death. The mouse macrophage cell line, RAW264.7, was also killed by a selective inhibitor of TAK1, 5z-7oxozeanol^38^ (Figure 4g, right graph). In RAW264.7 cells, inhibition of cathepsin B alone did not alter cell viability but partially rescued TAK1 inhibitor-induced cell death (Figure 4g). Taken together, these results demonstrate that *Tak1* deficiency impairs lysosomes, and that lysosomal dysfunction contributes to macrophage death.

### TNF is the trigger of cell death

**Figure 5:**
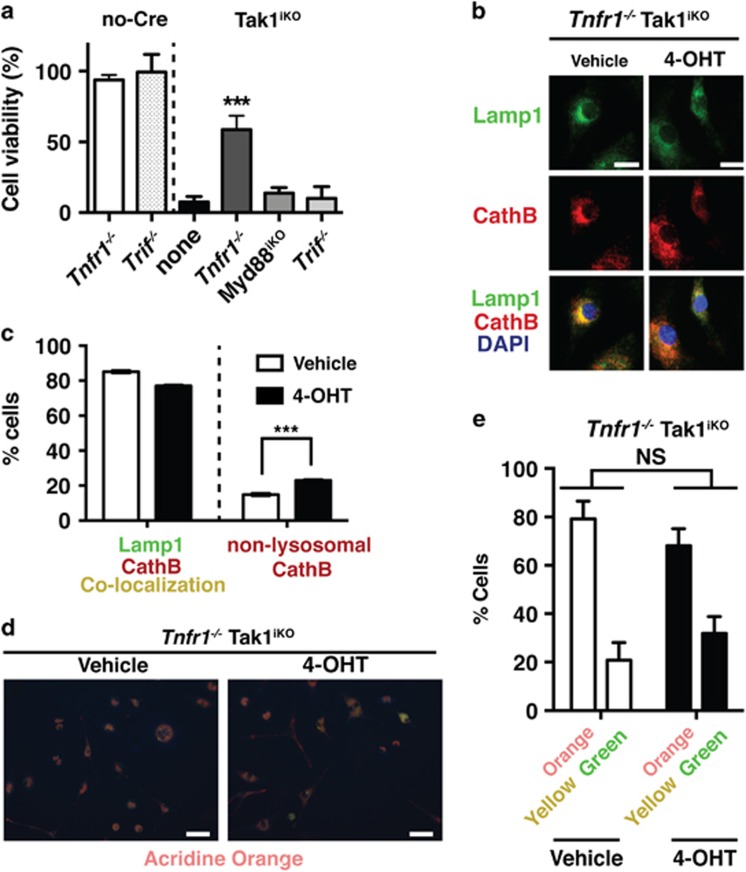
*Tnfr1* deletion partially protects lysosome and blocks cell death in *Tak1*-deficient BMDMs. (**a**) Percent cell viability of Tak1^iKO^ BMDMs in *Tnfr1*^−/−^ (*n*=3), *Myd88*^*iKO*^(*n*=3), or *Trif*^−/−^(*n*=3) backgrounds. Crystal violet stained 4-OHT-treated cells relative to those of the vehicle-treated same genotype cells were calculated and shown as percentages. Means±S.D.; ***P<0.001 (one-way ANOVA). (**b** and **c**) Tak1^iKO^ *Tnfr1*^−/−^ and no-Cre *Tnfr1*^−/−^ BMDMs were treated with vehicle or 0.3 *μ*M 4-OHT for 5 days. Lysosomal architecture was visualized by staining using anti-lamp1 (green) and anti-cathepsin B (CathB; red) antibodies. Scale bar, 10 *μ*m. Means±S.D.; ***P<0.001 (one-way ANOVA). (**d** and **e**) Lysosomal function was determined by incubating cells in acridine orange at 4 days post 4-OHT treatment. Orange staining indicates normal functional lysosomal pH (around 3.5), and yellow or green staining indicates increased lysosomal pH. Scale bar, 50 *μ*m. Means±S.D.; ***P<0.001 (one-way ANOVA)

*Tak1*-deficient BMDMs die without any exogenous stimuli in standard culture conditions. TAK1 is activated by a number of stressors including inflammatory cytokines and TLR ligands including dead cell-derived molecules (i.e., DAMPs). Macrophages produce inflammatory cytokines, TNF and IL-1, and dead cells are unavoidably present in the medium during isolation and culture of BMDMs. We asked whether TNF, IL-1 or TLR signaling is involved in *Tak1*-deficient BMDM death. To examine the TNF pathway, we utilized TNF receptor 1 (TNFR1)-deficient (*Tnfr1*^−/−^) mice.^39^ IL-1 and TLR pathways share the same adaptor MyD88, and some TLRs additionally utilize another adaptor, TRIF.^40^ To examine these pathways, we generated *Rosa26-CreERT Tak1*^flox/flox^ *Myd88*^flox/flox^ and *Rosa26-CreERT Tak1*^flox/flox^ *Trif*^−/−^ mice. An earlier study using the *LysM-Cre* system reported that TNF receptor deficiency rescues macrophage differentiation in myeloid-specific *Tak1* deletion bone marrow cells.^35^ However, involvement of TNF in macrophage viability after the completion of differentiation was not clear. We found that *Tnfr1* deficiency effectively albeit incompletely rescued Tak1^iKO^ BMDM death (Figure 5a), whereas deletion of either *Myd88* or *Trif* did not restore cell viability (Figure 5a). This suggests that TNF is, at least in part, the cause of Tak1^iKO^ BMDM death. Furthermore, we found that only a small proportion of cells exhibited abnormal localization of cathepsin B in the *Tnfr1*-deficient background (Figure 5b). Non-functional lysosomes with elevated pH were not increased by *Tak1* deletion on the *Tnfr1*^−/−^ background (Figures 5c and d). Collectively, these results suggest that TNF is the major cause of lysosomal dysfuntion, and associated cell death, in *Tak1*-deficient BMDMs.

### *Tnfr1* deletion partially rescues developmental abnormalities and loss of resident macrophages

**Figure 6:**
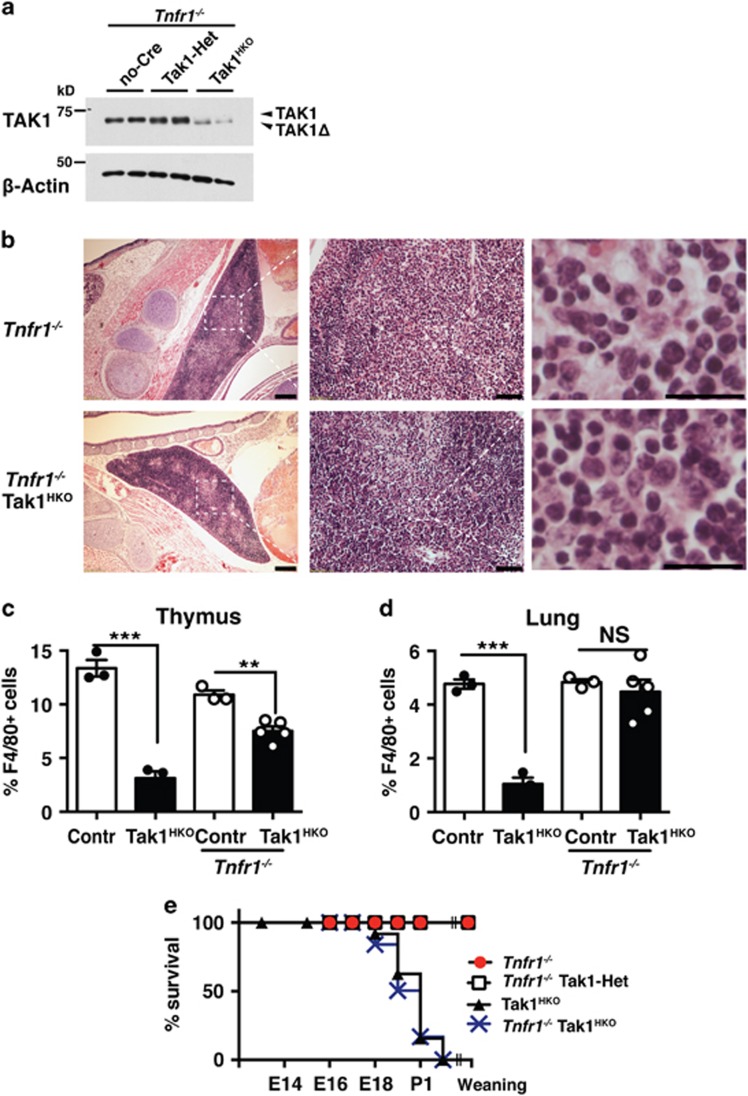
*Tnfr1* deletion partially restores tissue-resident macrophages and developmental abnormalities. (**a**) no-Cre *Tnfr1*^−/−^, Tak1-Het *Tnfr1*^−/−^, and Tak1^HKO^ *Tnfr1*^−/−^ thymocytes were isolated from E18.5 mice and analyzed by immunoblotting. Recombined *Tak1* gene expressed a truncated and non-functional TAK1 protein (TAK1Δ). *β*-actin is shown as a loading control. (**b**) H&E staining of E18.5 *Tnfr1*^−/−^ and Tak1^HKO^ *Tnfr1*^−/−^ thymus. Scale bars, 200 *μ*m (left panels), 50 *μ*m (middle panels), 20 *μ*m (right panels). (**c** and **d**) Quantification of F4/80^+^ cells in DAPI stained cells in control (Contr) and Tak1^HKO^ in wild type (left two bars) and *Tnfr1*^−/−^background (right two bars). The left two bars are the same data shown in Figures 3e and g. (**c**) P0 *Tnfr1*^−/−^ (*n*=3) and Tak1^HKO^ *Tnfr1*^−/−^ (*n*=3) thymus; and (**d**) P0 *Tnfr1*^−/−^ (*n*=3) and Tak1^HKO^ *Tnfr1*^−/−^ (*n*=5) lungs. Means±S.E. and all data points are shown. ***P<0.001; NS, not significant (two-tailed Student's *t-*test with equal distributions). (**e**) Percentages of viable *Tnfr1*^−/−^, Tak1-Het *Tnfr1*^−/−^, and Tak1^HKO^ *Tnfr1*^−/−^ mice. Viability data of Tak1^HKO^ (Figure 1a) are also included as a comparison. The total numbers of animals analyzed including live and dead were *Tnfr1*^−/−^, 100; Tak1-Het *Tnfr1*^−/−^, 51, Tak1^HKO^ *Tnfr1*^−/−^, 48; *P*<0.0001 (log-rank test)

We finally examined whether TNF-induced cell death is the cause of diminished resident macrophages in Tak1^HKO^ mice. We generated and analyzed Tak1^HKO^ *Tnfr1*^−/−^ mice. *Tnfr1*^−/−^ mice are normal under standard housing conditions, and there is no overt abnormality in morphogenesis.^41^ TAK1 was effectively depleted in the Tak1^HKO^ *Tnfr1*^−/−^ thymus at P0 (Figure 6a), similar to Tak1^HKO^ shown in Figure 1b. However, the cell debris, which was profoundly observed in the Tak1^HKO^ thymus (Figure 1e), was not seen in the Tak1^HKO^ *Tnfr1*^−/−^ thymus (Figure 6b). The number of thymic macrophages was still reduced in the Tak1^HKO^ *Tnfr1*^−/−^ thymus (Figure 6c), but it was improved compared with those in Tak1^HKO^ thymus (Figures 3d and f). Furthermore, the number of pulmonary macrophages was restored by *Tnfr1* deficiency (Figure 6d). These results demonstrate that, although other unidentified causes contribute to reduction of *Tak1*-deficient macrophages, TNF is one of the major triggers of diminished resident macrophages in Tak1^HKO^ mice. However, we note that animal mortality was not rescued by *Tnfr1* deletion (Figure 6e), suggesting that reduction of macrophages is not the prominent cause of animal mortality, and also that the mortality is TNF-independent. Collectively, although we could not identify the cause of animal mortality, our results reveal that TAK1 is a critical regulator of macrophage maintenance in the perinatal hematopoietic system by preventing TNF-induced lysosomal damage.

## Discussion

TAK1 is a signaling molecule that both promotes inflammatory responses and guards against cell death during inflammation in several cell types. We previously reported that *Tak1*-deficient epithelial and endothelial cells die, causing severe tissue damage.^6, 7, 8, 10, 11^ In the current study, we show that *Tak1* deficiency uniquely causes cell death in macrophages among hematopoietic cells during embryogenesis. Although *Tak1*-deficient thymocytes, splenocytes and circulating myeloid cells develop normally, *Tak1* deficiency diminishes thymic and lung-resident macrophages. This raises a question as to why certain cell types are selectively sensitive to *Tak1* deficiency. One possibility is that proliferative cells may be sensitive to *Tak1* deficiency. This idea is consistent with the fact that constantly renewing tissues such as the epidermis and the intestinal epithelium are highly sensitive to *Tak1* deficiency,^7, 8^ whereas neurons, which are mostly post-mitotic, are resistant to *Tak1* deficiency.^13^ However, it is inconsistent with the fact that embryonic hematopoietic progenitors are highly proliferative but the hematopoietic system developed normally in Tak1^HKO^ mice. Furthermore, cultured fibroblasts are also highly proliferative under standard culture conditions; however, *Tak1* deletion does not impair cell viability.^5^ In contrast, adult HSCs are known to be slowly self-renewing cells, but they are effectively depleted by *Tak1* gene deletion.^14^ Similarly, cultured macrophages (BMDMs) are mostly post-mitotic or very slow growing after completion of the differentiation processes, but they die upon *Tak1* gene deletion. Thus, cell proliferation is unlikely to be the determinant of the sensitivity to *Tak1* deficiency.

*Tak1*-deficient fibroblasts and keratinocytes undergo cell death when they are treated with TNF.^8, 42^ TNF treatment induces accumulation of ROS in *Tak1*-deficient cells. Similarly, ablation of *Tak1* activity in BMDMs highly upregulates ROS.^43^ TNF triggers cellular ROS production and phagocytic macrophages produce ROS.^44, 45^ Furthermore, ROS participate in adult HSC renewal and differentiation,^46, 47^ which may be further upregulated by *Tak1* deficiency. These issues raise the possibility that ROS may be the determinant of *Tak1* sensitivity. Although all cells constantly produce ROS as by-products of respiration, cells producing ROS beyond a certain level such as macrophages and TNF-treated fibroblasts may perhaps be killed if functional TAK1 is absent. We have previously reported that several cellular antioxidant enzymes such as glutamate-cysteine ligase catalytic subunit and NAD(P)H quinone dehydrogenase 1 and an antioxidant transcription factor Nrf2 are downregulated by *Tak1* deficiency.^10, 11, 12^ Although the molecular mechanism by which TAK1 regulates ROS is not fully understood, ablation of TAK1 seems to reduce the capacity of the cellular antioxidant system, resulting in accumulation of a cell-killing level of ROS if cells actively produce ROS.

Increased ROS are commonly and causally associated with cell death in *Tak1*-deficient keratinocytes, intestinal epithelial cells, and macrophages; however, the pathways of cell death vary depending on the cellular context. Caspase activity is profoundly upregulated in *Tak1*-deficient epidermis and intestinal epithelium as well as TNF-treated *Tak1*-deficient keratinocytes and fibroblasts,^10, 11, 42^ indicating that they die predominantly through apoptosis. Earlier studies also implicate TAK1 in programmed necrosis, so-called necroptosis, in the intestinal epithelium and macrophages.^32, 35^ Here, we show that *Tak1* deficiency causes lysosomal abnormality in macrophages. ROS are implicated in many types of cells death including apoptosis, necroptosis, and lysosomal rupture.^48, 49, 50^ ROS could trigger apoptosis through activation of mitochondrial membrane permeabilization and ROS-induced necroptotic protein assembly in macrophages. Our results demonstrate that apoptosis and necroptosis are not major forms of *Tak1*-deficient macrophage death. Phagocytic macrophages possess highly active phagosome-lysosomes, and lysosomal enzymes are highly expressed in macrophages. It is therefore possible that, due to such high lysosomal activity, *Tak1* deficiency may predominantly cause lysosomal damage through accumulation of ROS in macrophages but not other cell types.

Tissue-resident macrophages support tumor growth by clearing undesired molecules, supplying growth factors, and inducing angiogenesis.^51^ Thus, the cell type specific sensitivity to *Tak1* deficiency may be useful for developing approaches to manipulate macrophages in tumors. However, inhibition of TAK1 activity such as application of pharmacological inhibitors of TAK1 is anticipated to cause a number of undesired consequences in normal tissues based on the mouse studies using tissue-specific deletion of *Tak1*. The potential deleterious conditions include epithelial tissue damage^7, 8^ and skewing of adaptive immune cell populations,^16, 17, 18, 19^ which could lead to chronic inflammatory diseases if inhibition of TAK1 is prolonged. In contrast to prolonged inhibition of TAK1, temporary inhibition or inducible deletion of *Tak1* gene has thus far exhibited promising outcomes. Epidermal-specific inducible *Tak1* deletion can induce tumor regression in skin papilloma but does not cause observable injury in the normal skin.^9^ Treatment with a selective TAK1 inhibitor 5z-7oxozeanol^38^ effectively blocks tumor growth without overt toxicity.^52^ Thus, although inhibition of TAK1 must be entertained with considerable caution, regulated inhibition of TAK1 may be potentially useful to selectively kill macrophages without affecting adaptive immunity in certain contexts.

## Materials and Methods

### Mice

*Tak1*-floxed (*Tak1*^flox/flox^) mice have been described previously,^17^ and were backcrossed at least seven times to C57BL/6. *Vav-Cre* (Jax mice, B6.Cg-Tg(Vav1-icre)A2Kio/J),^26, 53^ *Rosa26-CreERT* (Jax mice, B6;129-Gt(ROSA)26Sortm1(cre/ERT)Nat/J),^36^ *Tnfr1*^−/−^ (B6.129-Tnfrsf1atm1Mak/J)^39^ and *Ripk3*^−/−^^54^ mice were bred in our facility to produce the genotypes used. For characterization of Tak1^HKO^ (*vav-Cre Tak1*^flox/flox^), littermate and wild type (no-Cre *Tak1*^flox/flox^) and heterozygous *Tak1* deletion (*vav-Cre Tak1*^flox/+^) mice, which were phenotypically indistinguishable, were used as controls. *Rosa26-CreERT Tab2*^flox/flox^ BMDMs were also used as controls, which were described previously.^34, 55^ Animal viability was calculated based on the assumption that Tak1^HKO^ mice were born at a Mendelian ratio. All animal experiments were conducted with the approval of the North Carolina State University Institutional Animal Care and Use Committee.

### Antibodies and reagents

TAK1,^56^ F4/80 (BM8, eBioscience, San Diego, CA, USA), CD16/32 (93, BioLegend, San Diego, CA, USA), CD4 (RM4-5, BioLegend), CD8a (53-6.7, BioLegend), CD11b (M1/70, BioLegend), Ly6C (HK1.4, BioLegend), Ly6G (1A8, BioLegend), cathepsin B (FL-339, Santa Cruz, Dallas, TX, USA), Lamp1 (Anti-Human CD107a, eBioscience or H4A3, Santa Cruz), and *β*-actin (AC-15, Sigma, St. Louis, MO, USA) antibodies were used. Human recombinant TNF*α* (Peprotech, Rocky Hill, NJ, USA), Z-VAD-FMK (Z-VAD) (Enzo Life Sciences, Farmingdale, NY, USA) and acridine orange (ThermoFisher Scientific, Waltham, MA, USA) were used. The TAK1 kinase inhibitor, 5Z-7-oxozeaenol (5Z) was described previously.^38^

### Histology and immunofluorescence staining of embryos

Embryo and neonate fixation and staining were performed using the method described previously.^57^ Briefly, E18.5 and P0 mice were killed, blanched in boiling water for 20 s, and immersed in an ice water bath to permit the removal of the epidermis. An incision was also made from the right clavicle to the pubic bone, opening the thoracic and abdominal cavities for improved penetration of fixative and processing reagents. Animals were fixed in Bouin's solution for 48 h at room temperature on a shaker followed by multiple 70% ethanol washes for 30 min each to remove excess fixative from the tissue before histiologic processing. E18.5 embryos from one litter consisting of three Tak1^HKO^ and three controls (no-Cre and heterozygous deletion) were step-sectioned dorsal to ventral at 300 *μ*ms, six serial sections (6 *μ*ms) were collected were stained with haemotoxylin and eosin, and the sections were pathologically evaluated by two board certified pathologists. For immunofluorescence staining of F4/80, 4% paraformaldehyde fixed embryo tissues were embedded in optimum cutting temperature (OCT) media, and 4–6 *μ*m cryosections were stained using anti-F4/80 (1 : 300) overnight at 4 °C. Bound antibodies were visualized by the Alexa Fluor 594 and 488 fluorescence dye-conjugated secondary antibodies (ThermoFisher Scientific). For quantification, more than 10 randomly digital images from each sample with the same exposure time were used.

### Flow cytometric analysis

Whole blood, thymus and spleen cells were isolated with Hank's balanced salt solution without magnesium and calcium (HBSS(−)). The cells were then suspended in 0.83% ammonium chloride for 5 min at room temperature to lyse red blood cells and washed with HBSS(−). Cells were resuspended in HBSS(−) and filtered to obtain single cell suspension, which was confirmed by a pulse geometry gate, FSC-A *versus* FSC-H. The cells were incubated for 20 min on ice with anti-CD16/32 antibody to block Fc*γ*RII/III, followed by incubation with fluorochrome-conjugated antibodies against cell surface antigens as described above. After labeling, cells were washed once with HBSS(−), resuspended in HBSS(−) and analyzed on FACS LSRII (BD Biosciences, Franklin Lakes, NJ, USA) and FlowJo (Version 10.1, FlowJo.LLC, Ashland, OR, USA). Debris was removed using a standard FSC *versus* SSC gating, and the subsequent gating strategy was shown in Figure 2.

### BMDMs and RAW264.7 cells

Bone marrow cells from indicated genotypes, Tak1^iKO^ (*Rosa26-CreERT Tak1*^flox/flox^) and other double deletion and littermate age-matched no-Cre control mice were isolated using a standard method and cultured in macrophage media containing 70% Dulbecco's modified Eagle's medium supplemented with 10% bovine growth serum (Hyclone) and 50 I.U./ml penicillin–streptomycin supplemented with 30% L929-conditioned media at 37 °C with 5% CO_2_. After 3-day culture, fully differentiated bone marrow-derived macrophages were re-plated and treated with 0.3 *μ*M 4-hydroxytamoxifen (4-OHT) or vehicle (ethanol) alone for 2–5 days to achieve gene deletion. Mouse macrophage RAW264.7 cells were cultured in Dulbecco's modified Eagle's medium supplemented with 10% bovine growth serum (Hyclone, San Angelo, TX, USA) and 50 I.U./ml penicillin–streptomycin.

### Crystal violet assay

BMDMs were plated onto 12- or 24-well plates and treated with 0.3 *μ*M 4-OHT or vehicle for 2 days and Z-VAD-FMK, Z-VAD (20 *μ*M), or TNF (20 ng/ml) for 3 additional days. RAW264.7 cells, cells were pre-treated with inhibitors and subsequently treated with 5Z-7 oxozeaenol. Cells were fixed using 10% formalin, and stained with 0.1% crystal violet solution. The dye was eluted and analyzed at 595 nm.

### Western blotting

BMDMs were lysed in extraction buffer (20 mM HEPES (pH 7.4), 150 mM NaCl, 12.5 mM *β*-glycerophosphate, 1.5 mM MgCl_2_, 2 mM EGTA, 10 mM NaF, 2 mM DTT, 1 mM Na_3_VO_4_, 1 mM PMSF, 20 *μ*M aprotinin, 0.5% Triton X-100, 50 nM Calyculin A) and incubated on ice for 30 min. Cell extracts were resolved using SDS-PAGE and transferred to Hybond-P membranes (GE Healthcare, Chicago, IL, USA). The membranes were immunoblotted with the indicated antibodies, and the bound antibodies were visualized with horseradish peroxidase-conjugated antibodies using the ECL (GE Healthcare) or SuperSignal West Femto (ThermoFisher Scientific).

### Immunofluorescence staining of BMDMs

BMDMs were seeded on glass coverslips in 6-well plates, were fixed with 4% paraformaldehyde for 10 min, and were permeabilized with 0.5 % Triton X-100 in PBS for 10–30 min at room temperature. The fixed cells were blocked with PBS containing 3% bovine serum albumin for 30 min at room temperature, and then incubated with anti-Lamp1 (1:100) and anti-cathepsin B antibodies (1:300) followed by incubation with anti-rat and anti-rabbit IgG conjugated with Alexa 594 or Alexa 488 (1:500, ThermoFisher Scientific). The coverslips were mounted with 50% glycerol and were examined by a fluorescence microscope (model BX41; Olympus, Tokyo, Japan) and camera (model DP80; Olympus) at room temperature. For quantification, more than 10 randomly photographed pictures from each sample with the same exposure time were used.

### Acridine orange staining

BMDMs were plated and incubated with 0.3 *μ*M 4-OHT or vehicle for 2–5 days, and stained for 15 min with 10 *μ*g/ml acridine orange in 1 × PBS. The images were taken using a UV filter (excitation 450–480 nm). For quantification, more than 10 randomly photographed pictures from each sample with the same exposure time were used.

### Statistical analysis

All experiments were conducted using at least three mice as indicated in figure legends and the results are confirmed by at least three separately performed experiments. The column graphs represent the mean±S.D. or S.E. as indicated. For data using *in vivo* samples, all data points are shown. Differences between experimental groups were assessed for significance by using the one-way ANOVA with Tukey's multiple comparisons test, or the unpaired Student's *t*-test (two-tailed) with equal distributions. For survival assay, the log-rank (Mantel–Cox) test was used.
