# Supplementary material for: TAK1 regulates resident macrophages by protecting lysosomal integrity
Source: Cell Death Dis. 2017 Feb 9;8(2):e2598–. doi: 10.1038/cddis.2017.23 (PMC5386472; doi:10.1038/cddis.2017.23)
Supplement: Supplementary Figures [file cddis201723x1.pdf]

Supplementary Materials

TAK1 Regulates Thymus Resident Macrophages By Protecting Lysosomal Integrity

Sakamachi Y. et al.

Supplementary Figures

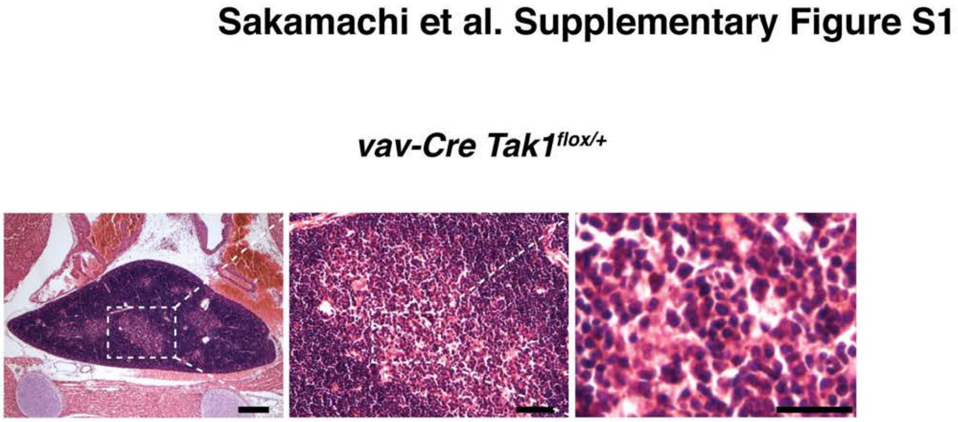

Figure S1. Hematopoietic-specific heterozygous deficiency of *Tak1* does not cause abnormality in the thymus

H&E staining of E18.5 *vav-Cre Tak1*$^{flox/+}$ thymus. Scale bars, 200 µm (left panel), 50 µm (middle panel), 20 µm (right panel).

Sakamachi et al. Supplementary Figure S2

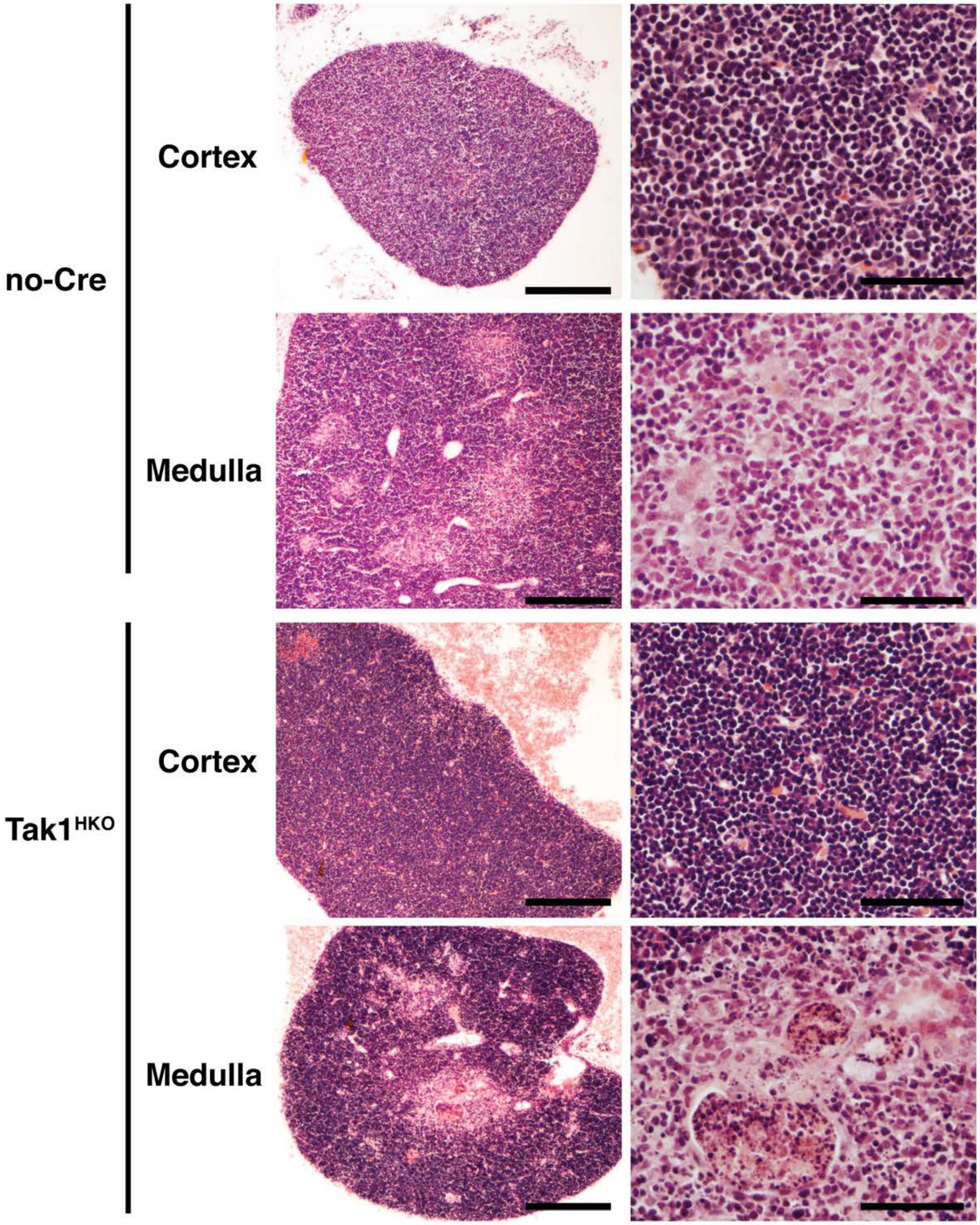

Figure S2. H&E staining of thymus
H&E staining of P0 no-Cre and Tak1^{HKO} thymus. Two different positions (cortex and medulla) from one thymus of no-Cre or Tak1^{HKO} mouse are shown. Scale bars, 200 µm (left panels), 50 µm (right panels).

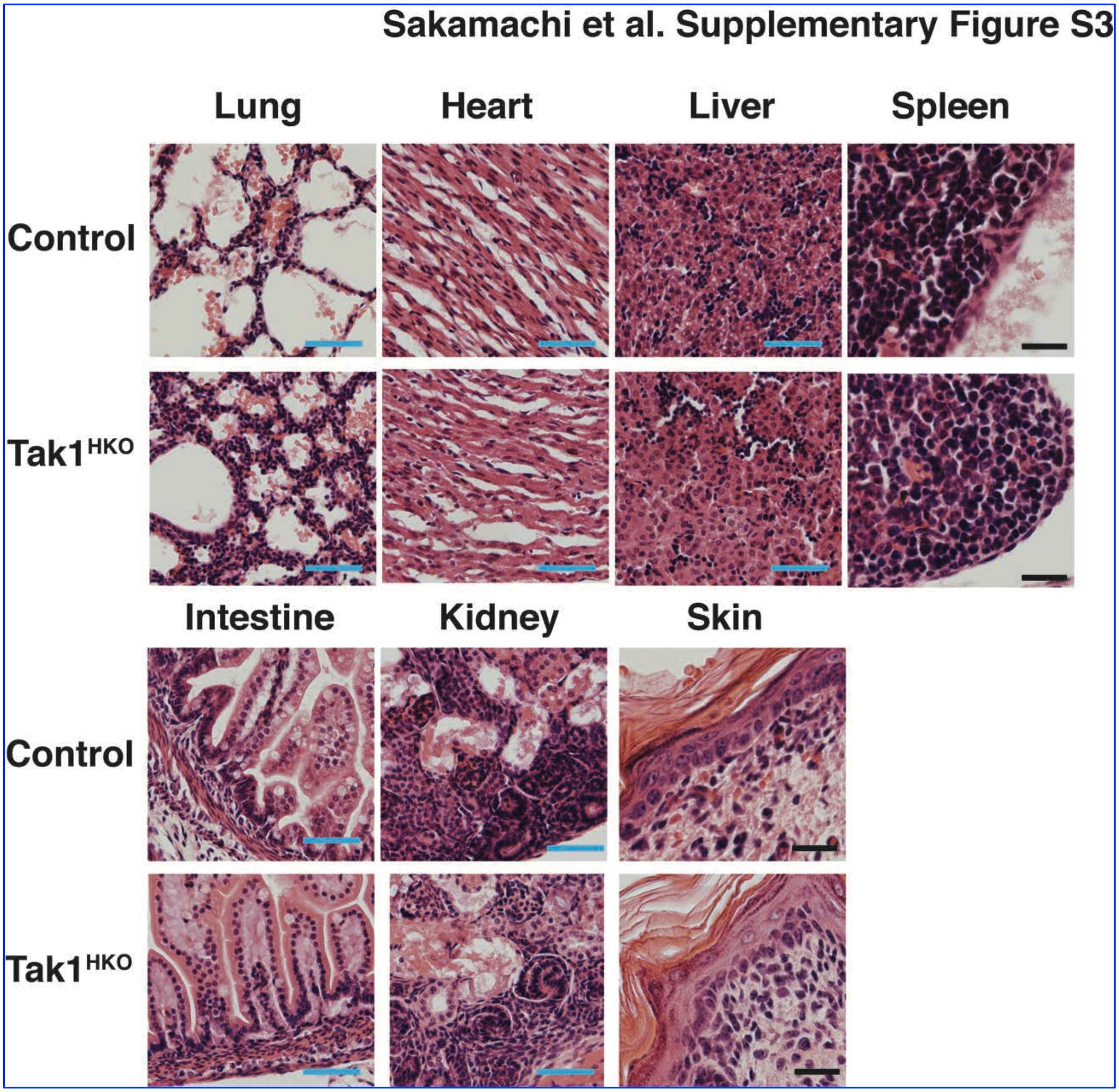

Figure S3. H&E staining of the lung, heart, liver, spleen, intestine, kidney and skin. Five controls including no-Cre and Tak1-Het and three Tak1^{HKO} mice at P0 were observed. Representative H&E staining images of control and Tak1^{HKO} tissues are shown. Scale bars, 50 µm (blue), 20 µm (black). The Tak1^{HKO} lungs exhibited impaired inflation, while other tissues of control and Tak1^{HKO} were indistinguishable.

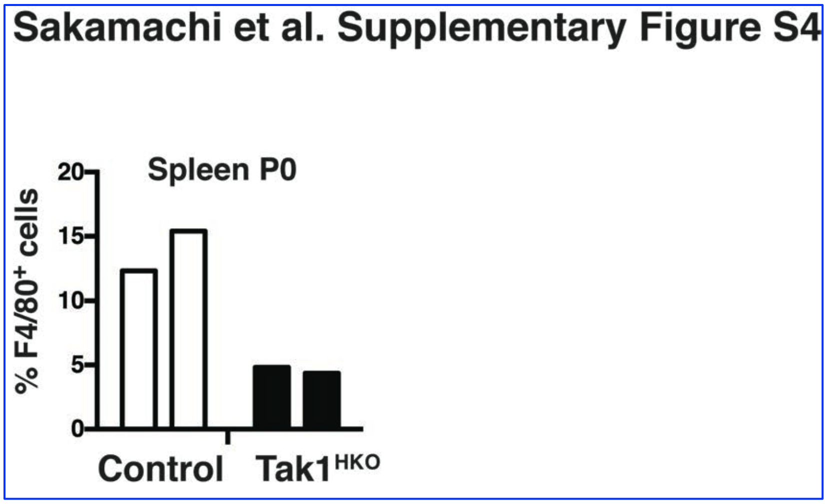

Figure S4. Spleen macrophages are diminished by *Tak1* deletion.
Control and Tak1^{HKO} spleens (n = 2 each) at P0 were analyzed by immunofluorescence staining using anti-F4/80 antibody (macrophages) and DAPI (all nuclei). F4/80^{+} cells in DAPI stained cells (more than 800 nuclei per animal form at least three randomly chosen images) were quantified.

Sakamachi et al. Supplementary Figure S5

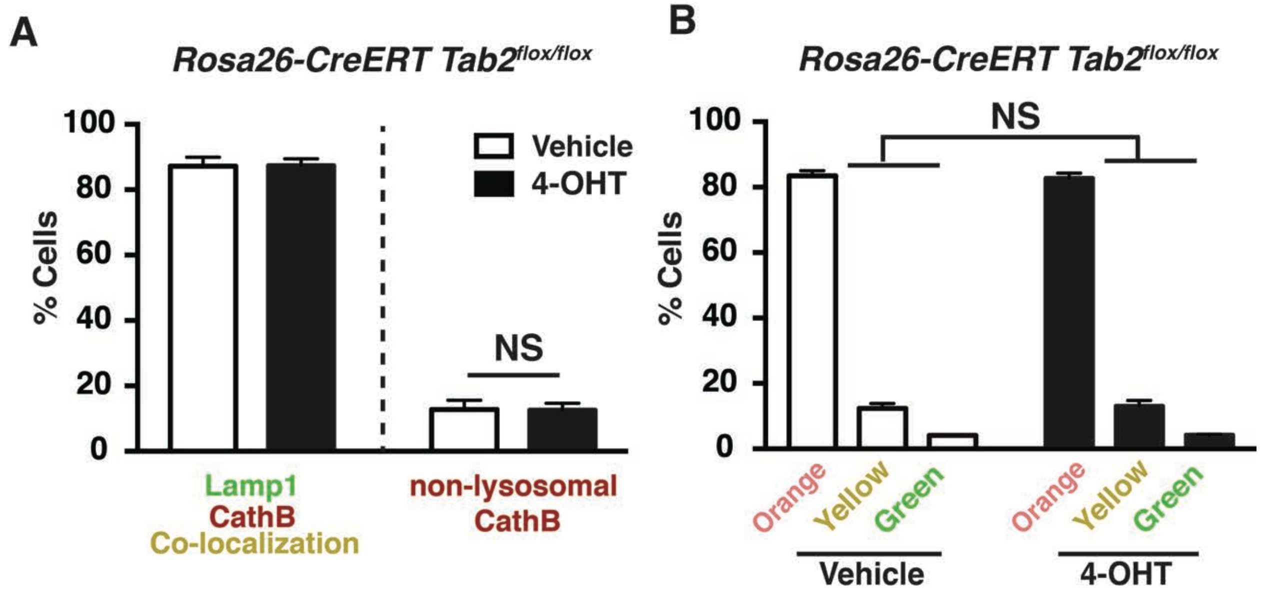

Figure S5. 4-OHT or Cre expression does not cause lysosomal injury
Rosa26-CreERT Tab2$^{flox/flox}$ BMDMs were treated with vehicle or 0.3 µM 4-OHT for 5 days. (A) Lysosomal architecture was visualized by staining using anti-lamp1 and anti-cathepsin B (CathB; red) antibodies, and quantified. Means ± SD; NS, not significant (unpaired two tailed Student-t test). (B) Lysosomal function was determined by incubating cells in acridine orange at 4 days post 4-OHT treatment. Orange staining indicates normal functional lysosomal pH (around 3.5), and yellow or green staining indicates increased lysosomal pH. Means ± SD; NS, not significant (one-way ANOVA).
